# Threshold selection and trimming in extremes

**DOI:** 10.1007/s10687-020-00385-0

**Published:** 2020-07-14

**Authors:** Martin Bladt, Hansjörg Albrecher, Jan Beirlant

**Affiliations:** 1 Department of Actuarial Science, Faculty of Business and Economics, University of Lausanne, CH-1015 Lausanne, Switzerland; 2 Department of Actuarial Science, Faculty of Business and Economics and Swiss Finance Institute, University of Lausanne, CH-1015 Lausanne, Switzerland; 3 Department of Mathematics, KU Leuven, Celestijnenlaan 200B, B-3001 Leuven, Belgium; 4 Department of Mathematical Statistics and Actuarial Science, University of the Free State, Bloemfontein, South Africa

**Keywords:** Trimming, Threshold selection, Regular variation, Hall class, 62F07, 62P05

## Abstract

We consider removing lower order statistics from the classical Hill estimator in extreme value statistics, and compensating for it by rescaling the remaining terms. Trajectories of these trimmed statistics as a function of the extent of trimming turn out to be quite flat near the optimal threshold value. For the regularly varying case, the classical threshold selection problem in tail estimation is then revisited, both visually via trimmed Hill plots and, for the Hall class, also mathematically via minimizing the expected empirical variance. This leads to a simple threshold selection procedure for the classical Hill estimator which circumvents the estimation of some of the tail characteristics, a problem which is usually the bottleneck in threshold selection. As a by-product, we derive an alternative estimator of the tail index, which assigns more weight to large observations, and works particularly well for relatively lighter tails. A simple ratio statistic routine is suggested to evaluate the goodness of the implied selection of the threshold. We illustrate the favourable performance and the potential of the proposed method with simulation studies and real insurance data.

## Appendix: Proofs

### **Proof of Proposition 2.1.**

Set *q* = *k* − *b* + 1. By the Rényi representation Eq. 6,

$$ \begin{array}{@{}rcl@{}} \mathbb{V}\left[T_{b,k}\right]&=\mathbb{V}\left[{\sum}_{j=1}^{k} E_{j}^{\ast} {\sum}_{i=j\vee q}^{k}\frac{\gamma_{i}}{k-j+1}\right]=\xi^{2} \frac{{\sum}_{j=1}^{k}\left( \frac{k-j\vee q+1}{k-j+1}\right)^{2}}{\left( {\sum}_{j=1}^{k}\frac{k-j\vee q+1}{k-j+1}\right)^{2}}. \end{array} $$

Plugging in *q* = 1 (*b* = *k*) gives

$$ \begin{array}{@{}rcl@{}} \mathbb{V}\left[T_{k,k}\right]=\frac{\xi^{2}}{k}, \end{array} $$

which corresponds to the usual variance of the Hill estimator *T*_*k*,*k*_ and gives the first identity. In the general case,

$$ \begin{array}{@{}rcl@{}} \mathbb{V}\left[T_{b,k}\right] &=\xi^{2} \frac{{\sum}_{j=1}^{q}\left( \frac{k- q+1}{k-j+1}\right)^{2}+k-q+1}{\left( {\sum}_{j=1}^{q}\frac{k- q+1}{k-j+1}+k-q+1\right)^{2}}. \end{array} $$

But *j* ≤ *q* implies $\frac {k-q+1}{k-j+1}\le 1$, such that

$$ \sum\limits_{j=1}^{q}\frac{1}{k-j+1}\ge \sum\limits_{j=1}^{q}\frac{k-q+1}{k-j+1}\frac{1}{k-j+1},$$

so

$$ \begin{array}{@{}rcl@{}} \sum\limits_{j=1}^{q}\frac{k- q+1}{k-j+1}+k-q+1\ge \sum\limits_{j=1}^{q}\left( \frac{k- q+1}{k-j+1}\right)^{2}+k-q+1. \end{array} $$

Thus

$$ \begin{array}{@{}rcl@{}} \mathbb{V}\left[T_{b,k}\right]&\le&\xi^{2}\frac{{\sum}_{j=1}^{q}\left( \frac{k- q+1}{k-j+1}\right)^{2}+k-q+1}{\left( {\sum}_{j=1}^{q}\left( \frac{k- q+1}{k-j+1}\right)^{2}+k-q+1\right)^{2}}\\ &=&\frac{\xi^{2}}{{\sum}_{j=1}^{q}\left( \frac{k- q+1}{k-j+1}\right)^{2}+k-q+1}, \end{array} $$

which gives the second identity. □

### **Proof of Theorem 3.1.**

We first note that

$$ \begin{array}{@{}rcl@{}} T_{b,k}\stackrel{d}{=}\frac{{\sum}_{i=1}^{b}\log(U(Y_{n-i+1,n})/U(Y_{n-k,n}))}{b(1+{\sum}_{j=b+1}^{k}j^{-1})}, \end{array} $$

where *Y*_1,*n*_ < ⋯ < *Y*_*n*,*n*_ are the order statistics of a standard Pareto sample (the *ξ* = 1 case). Then, from the second order condition Eq. 14 we obtain that for *A* = *Y*_*n*−*k*,*n*_ and *x* = *Y*_*n*−*i*+ 1,*n*_/*Y*_*n*−*k*,*n*_, as $k,n,n/k\to \infty $,

$$ \begin{array}{@{}rcl@{}} T_{b,k}\stackrel{d}{=}\!\frac{\xi{\sum}_{i=1}^{b} \log(Y_{n-i+1,n}/Y_{n-k,n})+\frac{Q_{0}(Y_{n-k,n})}{p}{\sum}_{i=1}^{b}((Y_{n-i+1,n}/Y_{n-k,n})^{p}-1)(1+o_{p}(1))}{b(1+{\sum}_{j=b+1}^{k}j^{-1})}. \end{array} $$

But by the Rényi representation Eq. 6 of exponential order statistics, the first term is distributed as

$$ \begin{array}{@{}rcl@{}} \sum\limits_{i=1}^{b}\log(Y_{n-i+1,n}/Y_{n-k,n})\stackrel{d}{=}\sum\limits_{j=1}^{b} E_{j}+b\sum\limits_{j=b+1}^{k}E_{j}/j, \end{array} $$

where $E_{1},E_{2},\dots , E_{k}$ are i.i.d. standard exponential random variables. For the second term, by convergence to uniform random variables and a Riemann integral approximation, we get

$$ \begin{array}{@{}rcl@{}} \frac 1b \sum\limits_{i=1}^{b}((Y_{n-i+1,n}/Y_{n-k,n})^{p}-1)&&\stackrel{d}{\approx}\frac 1b \sum\limits_{i=1}^{b}(((k+1)/i)^{p}-1)\\ &&\approx\! \frac{k+1}{b}\!{\int}_{0}^{b/(k+1)}(u^{\!-p}-1)\mathrm{d} u = \frac{((k+1)/b)^{p}}{1-p} - 1, \end{array} $$

and since (1 − 1/*Y*_*n*−*k*,*n*_) is a uniform order statistic, we further get that

$$ \begin{array}{@{}rcl@{}} \frac{Q_{0}(Y_{n-k,n})}{Q_{0}(n/k)}\stackrel{P}{\to}1. \end{array} $$

Putting the three pieces together then establishes Eq. 15. □

### **Proof of Theorem 3.2.**

With the shortened notation, we write

27 $$ \begin{array}{@{}rcl@{}} T_{b,k}\stackrel{d}{=}\xi \frac{\overline E_{b}+{\sum}_{j=b+1}^{k}E_{j}/j}{1+{\sum}_{j=b+1}^{k}j^{-1}}+Q_{0}(n/k)c_{b,k,p}(1+o_{p}(1)), \end{array} $$

and by exchange of the order of summation, we can write

$$ \begin{array}{@{}rcl@{}} \overline T_{k}&&\stackrel{d}{=}\frac{\xi}{k} \sum\limits_{b=1}^{k}\frac{\overline E_{b}+{\sum}_{j=b+1}^{k}E_{j}/j}{1+{\sum}_{j=b+1}^{k}j^{-1}}+Q_{0}(n/k)\overline c_{k,p}(1+o_{p}(1))\\ &&=\frac{\xi}{k}\left[{\sum}_{j=1}^{k}E_{j}{\sum}_{b=j}^{k}\frac{1}{b(1+{\sum}_{j=b+1}^{k}j^{-1})}+{\sum}_{j=2}^{k} E_{j}{\sum}_{b=1}^{j-1}\frac {1}{j(1+{\sum}_{j=b+1}^{k}j^{-1})}\right]\\\ &&\quad+Q_{0}(n/k)\overline c_{k,p}(1+o_{p}(1))\\ &&=\frac{\xi}{k}{\sum}_{j=1}^{k}E_{j}\left[{\sum}_{b=j}^{k}\frac{1}{b(1+\log(k/b)}+{\sum}_{b=1}^{j-1}\frac {1}{j(1+\log(k/b)}\right](1+o(1))\\\ &&\quad+Q_{0}(n/k)\overline c_{k,p}(1+o_{p}(1)). \end{array} $$

Again, by Riemann integration we have that

$$ \begin{array}{@{}rcl@{}} \frac 1k {\sum}_{b=j}^{k}\frac{1}{(b/k)(1+\log(k/b))}\approx{\int}_{j/k}^{1}\frac{\mathrm{d} u}{u(1-\log(u))}=\log(1+\log(k/j)), \end{array} $$

and

$$ \begin{array}{@{}rcl@{}} {\sum}_{b=1}^{j-1}\frac {1}{j(1+\log(k/b)}\approx \frac k j{\int}_{0}^{j/k}\frac{\mathrm{d} u}{1-\log(u)}=\frac {ek}{j} \mathtt{E}(1+\log(k/j)). \end{array} $$

Similarly,

28 $$ \begin{array}{@{}rcl@{}} \overline c_{k,p}&\approx \frac 1p {{\int}_{0}^{1}} \frac{(1-p)^{-1}u^{-p}}{1-\log(u)}\mathrm{d} u-\frac 1 p {{\int}_{0}^{1}}\frac {\mathrm{d} u}{1-\log(u)}\\ &=\frac{e^{1-p}}{p(1-p)}\mathtt{E}(1-p)-\frac{e}{p}\mathtt{E}(1). \end{array} $$

Putting the pieces together then indeed yields Eq. 18. □

### **Proof of Theorem 3.3.**

Let us first decompose each summand by writing

$$ \begin{array}{@{}rcl@{}} \mathbb{E}[(T_{b,k}-\overline T_{k})^{2}]=\mathbb{E}[(T_{b,k}-\xi)^{2}]+\mathbb{E}[(\overline T_{k}-\xi)^{2}]-2\mathbb{E}[(T_{b,k}-\xi)(\overline T_{k}-\xi)], \end{array} $$

and subsequently consider each term separately. From Eq. 27 we have that

$$ \begin{array}{@{}rcl@{}} \mathbb{E}[(T_{b,k}-\xi)^{2}]&=\mathbb{V}[T_{b,k}]+\text{Bias}^{2}[T_{b,k}] &=\xi^{2} \frac{\frac{1}{b}+{\sum}_{j=b+1}^{k}1/j^{2}}{(1+{\sum}_{j=b+1}^{k}j^{-1})^{2}}+{Q_{0}^{2}}(n/k)c_{b,k,p}^{2}(1+o_{p}(1)). \end{array} $$

On the other hand, Eq. 18 gives

$$ \begin{array}{@{}rcl@{}} \mathbb{E}[(\overline T_{k}-\xi)^{2}]&=&\mathbb{V}[\overline T_{k}]+\text{Bias}^{2}[\overline T_{k}]\\ &=&\frac{\xi^{2}}{k^{2}}{\sum}_{j=1}^{k}\left[\log(1+\log(k/j))+\frac {ek}{j} \mathtt{E}(1+\log(k/j))\right]^{2}(1+o(1))\\ &&+ {Q_{0}^{2}}(n/k)\left[\frac{e^{1-p}}{p(1-p)}\mathtt{E}(1-p)-\frac{e}{p}\mathtt{E}(1)\right]^{2}(1+o_{p}(1)). \end{array} $$

The third term can be analyzed using both Eqs. 18 and 27 as follows,

$$ \begin{array}{@{}rcl@{}} &&\mathbb{E}[(T_{b,k}-\xi)(\overline T_{k}-\xi)]=\mathbb{E}[(T_{b,k}-\mathbb{E}[T_{b,k}])(\overline T_{k}-\mathbb{E}[\overline T_{k}])]+{Q_{0}^{2}}(n/k)c_{b,k,p}\overline c_{k,p}\\ &&=\xi^{2}\mathbb{E}\left[\left( \frac{\frac 1 b {\sum}_{j=1}^{b} (E_{j}-1)+{\sum}_{j=b+1}^{k}(E_{j}-1)/j}{1+{\sum}_{j=b+1}^{k}j^{-1}}\right) \left( \sum\limits_{i=1}^{k}\frac {E_{i} -1}{k} S(i,k)(1+o(1))\right)\right]\\ &&\quad+{Q_{0}^{2}}(n/k)c_{b,k,p}\overline c_{k,p}\\ &&=\xi^{2}\frac{{\sum}_{j=1}^{k} (j\vee b)^{-1} S(j,k)}{k(1+{\sum}_{j=b+1}^{k}j^{-1})}(1+o(1))+{Q_{0}^{2}}(n/k)c_{b,k,p}\overline c_{k,p} \end{array} $$

where $S(j,k):=\log (1+\log (k/j))+\frac {ek}{j} \mathtt {E}(1+\log (k/j))$.

We now proceed to add the *k* summands of the expected variance. To this end, some preparatory calculations will be helpful. By Eq. 17 and Riemann approximation we have

$$ \begin{array}{@{}rcl@{}} \frac{1}{k}\sum\limits_{b=1}^{k}c_{b,k,p}^{2}&\approx& \frac{1}{k}\sum\limits_{b=1}^{k}\frac{1}{p^{2}}\cdot \frac{\frac{((k+1)/b)^{2p}}{(1-p)^{2}}-2\frac{((k+1)/b)^{p}}{1-p}+1}{(1+\log((k+1)/b))^{2}}\\ &\approx& \frac{1}{p^{2}(1-p)^{2}}\left[1-e^{1-2p}(1-2p)\mathtt{E}(1-2p)\right]\\ && -\frac{2}{p^{2}(1-p)}\left[1-e^{1-p}(1-p)\mathtt{E}(1-p)\right]\\ && +\frac{1}{p^{2}}\left[1-e\mathtt{E}(1)\right]. \end{array} $$

By virtue of Eq. 28,

$$ \begin{array}{@{}rcl@{}} \overline c_{k,p}^{2} \approx \frac{e^{2(1-p)}}{p^{2}(1-p)^{2}}\mathtt{E}^{2}(1-p)-2 \frac{e^{2-p}}{p^{2}(1-p)}\mathtt{E}(1-p)\mathtt{E}(1)+\frac{e^{2}}{p^{2}}\mathtt{E}^{2}(1), \end{array} $$

from which we deduce that as $k\to \infty $,

$$ \begin{array}{@{}rcl@{}} \frac{1}{k}\sum\limits_{b=1}^{k}c_{b,k,p}^{2}-\overline c_{k,p}^{2}\to f(p), \end{array} $$

where *f*(*p*) is given by Eq. 19.

Observe that for the terms arising from the expected covariance term

$$ \begin{array}{@{}rcl@{}} \frac{1}{k}\sum\limits_{b=1}^{k}\frac{\frac{1}{b}+{\sum}_{j=b+1}^{k}1/j^{2}}{(1+{\sum}_{j=b+1}^{k}j^{-1})^{2}}&&\approx\frac{2}{k}{{\int}_{0}^{1}}\frac{\mathrm{d} u}{u(1-\log(u))^{2}}-\frac{1}{k}{{\int}_{0}^{1}}\frac{\mathrm{d} u}{(1-\log(u))^{2}}\\ &&=\frac{1+e\mathtt{E}(1)}{k}. \end{array} $$

Next,

$$ \begin{array}{@{}rcl@{}} &&\frac{1}{e}\frac{1}{k}\sum\limits_{b=1}^{k}\frac{{\sum}_{j=1}^{b} b^{-1} (\log(1+\log(k/j))+\frac {ek}{j} { \mathtt{E}}(1+\log(k/j)))}{1+\log(k/b)}\\ &\approx& {{\int}_{0}^{1}}\frac{1}{z\log(e/z)}\left( {{\int}_{0}^{z}} \frac{1}{u} \left( {\int}_{\log(e/u)}^{\infty}\log(v)e^{-v}\mathrm{d} v\right)\mathrm{d} u\right) \mathrm{d} z\\ &\approx& 0.266=:I_{1} \end{array} $$

and

$$ \begin{array}{@{}rcl@{}} &&\frac{1}{e}\frac{1}{k}\sum\limits_{b=1}^{k}\frac{{\sum}_{j=b+1}^{k} j^{-1} (\log(1+\log(k/j))+\frac {ek}{j} { \mathtt{E}}(1+\log(k/j)))}{1+\log(k/b)}\\ &\approx& {{\int}_{0}^{1}}\frac{1}{\log(e/z)}\left( {{\int}_{z}^{1}} \frac{1}{u^{2}} \left( {\int}_{\log(e/u)}^{\infty}\log(v)e^{-v}\mathrm{d} v\right)\mathrm{d} u\right) \mathrm{d} z\\ &\approx&0.135746=:I_{2}. \end{array} $$

Finally, for the mean squared error term of $\overline {T}_{k}$ we find

$$ \begin{array}{@{}rcl@{}} &\frac{1}{k}\sum\limits_{j=1}^{k}(k/j)^{2}\left( {\int}_{\log(e/u)}^{\infty} \log(v)e^{-v}\mathrm{d} v\right)^{2}\\ &{\approx{\int}_{0}^{1}}u^{-2}\left( {\int}_{\log(e/u)}^{\infty} \log(v)e^{-v}\mathrm{d} v\right)^{2} \mathrm{d} u\\ &\approx 0.148005=:I_{3}. \end{array} $$

Altogether we hence obtain

$$ \begin{array}{@{}rcl@{}} &&\mathbb{E}\left[\frac{1}{k}\sum\limits_{b=1}^{k}(T_{b,k}-\overline T_{k})^{2}\right]\\ &=&\frac{1}{k}\sum\limits_{b=1}^{k}\left( \mathbb{E}[(T_{b,k}-\xi)^{2}]+\mathbb{E}[(\overline T_{k}-\xi)^{2}]-2\mathbb{E}[(T_{b,k}-\xi)(\overline T_{k}-\xi)]\right)\\ &=&\xi^{2}\left( \frac{1+e\mathtt{E} (1)}{k}\right)(1+o(1))+\xi^{2}\frac{e^{2}}{k}I_{3}(1+o(1))\\ &&-2\xi^{2} \frac{e}{k}(I_{1}+I_{2})(1+o(1))+{Q_{0}^{2}}(n/k)\left[\frac{1}{k}\sum\limits_{b=1}^{k}c_{b,k,p}^{2}-\overline c_{k,p}^{2}\right](1+o_{p}(1))\\ &=&\xi^{2}\left( \frac{1+e\mathtt{E}(1)}{k}\right)(1+o(1))+\xi^{2}\frac{e^{2}}{k}I_{3}(1+o(1))\\ &&-2\xi^{2} \frac{e}{k}(I_{1}+I_{2})(1+o(1))+{Q_{0}^{2}}(n/k)f(p)(1+o_{p}(1))\\ &=&\frac{C}{k} \xi^{2}(1+o(1))+{Q_{0}^{2}}(n/k)f(p)(1+o_{p}(1)) \end{array} $$

with

$$ \begin{array}{@{}rcl@{}} C=1+e\mathtt{E}(1)+e^{2}I_{3}-2e(I_{1}+I_{2})\approx 0.502727. \end{array} $$

□
